# A Personalized, Dynamic Physical Activity Intervention Is Feasible and Improves Energetic Capacity, Energy Expenditure, and Quality of Life in Breast Cancer Survivors

**DOI:** 10.3389/fonc.2021.626180

**Published:** 2021-04-12

**Authors:** Tarah J. Ballinger, Sandra K. Althouse, Timothy P. Olsen, Kathy D. Miller, Jeffrey S. Sledge

**Affiliations:** ^1^ Department of Medicine, Indiana University School of Medicine, Indianapolis, IN, United States; ^2^ Department of Urban and Regional Planning, University of Wisconsin, Madison, WI, United States

**Keywords:** breast cancer, survivors, physical activity, activity trackers, accelerometry

## Abstract

**Purpose:**

Despite survival and quality of life benefits associated with physical activity, many breast cancer survivors remain inactive. Effective, sustainable interventions must account for individual differences in capability, motivation, and environment. Here, we evaluate the feasibility, mechanics, and efficacy of delivering an individualized, dynamic intervention to increase energetic capacity and energy expenditure.

**Methods:**

Stage 0–III breast cancer patients who had completed primary treatment were enrolled. Prior to the intervention, detailed movement data was collected with a wearable GPS and accelerometer for 3 weeks to establish baseline activity. Movement data was collected continuously throughout the 12-week intervention, during which patients received electronically delivered, tailored, dynamic activity “prescriptions”, adjusted based on demonstrated individual capability, daily movement in their environment, and progress.

**Results:**

Of 66 enrolled, 57 participants began and completed the intervention. The intervention resulted in significant improvements in average steps (+558 steps/day, p = 0.01), energetic capacity measured by power generation on a stationary bicycle (1.76 to 1.99 W/kg lean mass, p < 0.01), and quality of life (FACT-B TOI, 72.8 to 74.8, p = 0.02). The greatest improvement in functional energetic capacity was seen in the lowest performing tertile at baseline (0.76 to 1.12 W/kg, p < 0.01).

**Discussion:**

Wearable technology delivery of personalized activity prescriptions based on individual capability and movement behaviors demonstrates feasibility and early effectiveness. The high variability seen in baseline activity and function, as well as in response to the intervention, supports the need for future work in precision approaches to physical activity (NCT03158519).

## Introduction

Physical activity is significantly associated with improved overall health, quality of life, and disease specific survival outcomes in breast cancer (1, 2). Breast cancer treatment has a detrimental effect on functional capabilities, even years after completing treatment (3, 4). A significant proportion of women with a history of breast cancer do not meet physical activity guidelines, are less active than their peers unaffected by breast cancer, and self-report decreased physical activity levels following diagnosis (5, 6).

Exercise intervention trials in this population are feasible with modest effect sizes in several important endpoints, including body composition, metabolic syndrome, and quality of life (7, 8). However, individuals with limited exercise experience or higher levels of baseline fatigue are less likely to participate or comply with activity interventions (9–11). Thus, the population most in need is the least likely to benefit from current strategies involving intimidating, gym-based or one-size-fits-all approaches. The opportunity to be physically active is individual, depending on motivation (12, 13), environment (14, 15), and capability (16, 17). Effective activity interventions must take these differences into account and meet patients where they are, particularly for the most debilitated or inactive patients.

Here, we evaluate a personalized activity intervention, designed to allow patients to incrementally increase movement by leveraging their current ability and patterns of physical activity within their built environment. The intervention delivers dynamic physical activity prescriptions based on individual wearable data from continuous GPS, heart rate, and accelerometer monitoring. Rather than asking participants to begin new or foreign behaviors, this strategy leverages constructivist, conceptual change, and legitimate peripheral practice theories to ground physical activity instructions in what patients are already able to do and believe is worthwhile (18, 19). The objective of this pilot trial was to evaluate the retention, feasibility, and early effect of this approach on energetic capacity, movement (steps/day), and quality of life in breast cancer survivors.

## Methods

This prospective, observational, single arm study was designed to assess the feasibility and efficacy of a semi-automated, individualized physical activity intervention delivered *via* wearable, combined GPS/accelerometer in breast cancer survivors. Primary and secondary endpoints were measured before and after the 12-week intervention. The Indiana University Institutional Review Board approved the study and patients provided written informed consent prior to participation. Research was performed in accordance with the ethical standards of the Declaration of Helsinki.

### Study Participants

Eligible participants had a history of ductal carcinoma *in situ* (DCIS) or stage I–III invasive breast cancer and were recruited at the Indiana University Simon Cancer Center and Eskenazi Health county hospital in Indianapolis, Indiana. Rather than population-based or convenience sampling methods, recruitment was performed pragmatically in breast oncology clinics and any patient meeting the eligibility criteria was approached. Eligibility criteria included completing all primary therapy for breast cancer at least 4 weeks prior to enrollment, with the exception of ongoing endocrine or HER2 directed therapy. Body weight was required to be less than 300 pounds due to weight limit of the DEXA scanner. Subjects had no uncontrolled neurologic, orthopedic, cardiac, or pulmonary conditions that would interfere with the ability to safely ride a stationary bicycle. Participants were compensated $50 for each assessment and at weeks 4 and 8 during the intervention, for a total of $200, regardless of compliance.

### Study Design and Activity Intervention

Prior to the intervention, individual movement and energy expenditure data was passively collected for 3 weeks in order to establish baseline patterns of activity. During this period, enrolled patients wore a wrist GPS/accelerometer-enabled activity and heart rate monitor (Garmin Vivoactive HR®). This device provides valid and reliable measurements of heart rate and step count (20). GPS provided spatial location of the patient at individual time points. Combined with accelerometer and transdermal heart rate monitoring, as well as integration with community maps and weather, GPS data allowed energetic demands and energetic expenditure to be determined at a higher level of spatial accuracy than GPS or accelerometer alone. Subjects were asked to wear the GPS/accelerometer for all waking hours throughout the conduct of the study.

Following passive movement data collection, participants underwent baseline assessment of energetic capacity measured by power output on a stationary bicycle, body composition by DEXA, and questionnaires assessing fatigue and quality of life. A patient portal was downloaded onto the participant’s smartphone and subjects were shown how to sync wearable data. Access to either a smartphone, tablet, or computer with web access was required for study participation; subjects could access the portal by any of these means per preference.

**Figure 1:**
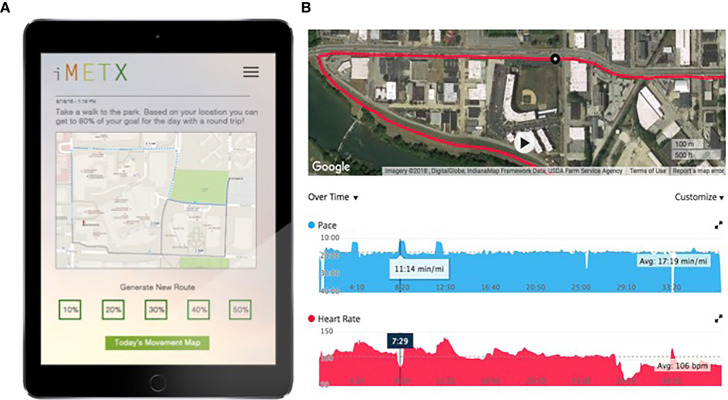
Example of patient interface and energy expenditure data. **(A)** displays patient view of movement prescription to incorporate bursts of more vigorous activity into her walks. **(B)** displays the same patient’s movement within her community, incorporating the prescription as evidenced by changes in her pace and heart rate.

During the 12-week intervention, the study investigators and web-based application utilized the subjects’ baseline energetic capacity determined from peak power output on the stationary bicycle and movement patterns derived from the GPS/accelerometer data to design daily activity “prescriptions”. The intervention was developed using a geospatial model within ArcGIS 10.X software. GPS-based, real-time community level maps were created displaying the potential energy expenditure of each participant within their community (**Figure 1**). Integration of heart rate data, steps taken, and the routes moved within the subject’s environment was used to generate a personal activity prescription for the following day, delivered to the patient portal. The initial prescription was based on the baseline movement data collected and peak power generated on a stationary bicycle at baseline (described below).

During the intervention, the prescribed daily movement objectives accounted for location, anticipated weather, and varying volumes and intensity of energy expended by the patient. Based on 3-day rolling average of energy expenditure data from the wearable, the activity prescriptions were adjusted up or down. In this way, the intervention was dynamic and could scale the activity prescribed up or down based on the patient’s ability to meet or exceed the prior prescription, always with the goal of increased activity. For example, if patients were not meeting the movement goals at the end of a 3-day average, the system automatically decreased the recommended individualized movement as a percentage below the previous recommendation. In this way, the algorithms are designed to responsively adjust goals based on patient performance by their wearable data, much as a personal trainer might. Heart rate data compared to activity level intensity was used as a safety factor and monitored changes in patient performance. Based on the flow of data from the wearable and system algorithms, the application “learned” to optimize how individual energetic demands were prescribed to maximize patient adaptation and produce higher levels of physical activity and capacity. Rather than simply tracking activity, the intervention responded to it in a personalized way that might be possible with an exercise trainer, if the trainer had the same access to the data and analytics, but was able to deliver prescriptions remotely and dynamically in real time. Following the 12-week intervention, participants repeated the stationary bicycle protocol and baseline questionnaires. As an exploratory evaluation of sustained behavior change, participants were asked to continue wearing the activity monitor for an additional 12 weeks in order to observe their behavior and begin exploring whether activity changes were sustained in the absence of active intervention.

### Outcome Measures

#### Feasibility

The primary objective was to evaluate the feasibility of this individualized, dynamic physical activity intervention. Feasibility was determined by the percentage of enrolled patients who completed all assessments and wore the GPS/accelerometer activity monitor for at least 10 h per day, 4 days a week, based on the NHANES standard for physical activity data completeness (21). Qualitative patient feedback was collected from participants throughout the intervention with a feedback area in the patient portal.

#### Steps/Day

The activity tracker incorporated both an accelerometer and GPS unit. The accelerometer recorded step counts and stair count along with intensity of movement. The GPS unit provided additional and complimentary data such that GIS data, steps, activity type (walking, running and stairs), and patient level data could be integrated to calculate energetic expenditure within the patient’s environment. Participants wore the activity tracker for a 3-week pre-intervention baseline period to define their habituated energy expenditure environment, and throughout the intervention to provide objective measures of step counts, movement patterns, and GIS derived measures of energy expenditure. Movement was assessed in near real-time during the intervention to inform activity prescriptions. Average daily step count during the intervention was compared to the pre-intervention period as a secondary endpoint.

#### Energetic Capacity

Energetic capacity was measured using the Power Protocol-B™, as previously described (17, 22). In brief, the Power Protocol-B™ is a stationary bicycle-based procedure that establishes the range of power performance of the subject by incrementally increasing power demands until stopping criteria are reached. This procedure was used as it is less invasive, costly, intimidating, or technically difficult than VO_2max_ testing and can be administered in the clinic by nursing staff. In addition, the Power-Protocol-B™ is independent of patient effort across a wide range of abilities. Ten second mean peak watts generated per kilogram of lean body mass (watts/kg) measured by DEXA was recorded pre- and post-intervention.

#### Anthropometrics/Dual-Energy X-Ray Absorptiometry (DEXA)

Weight was measured to 0.1 kg on an electronic scale and height was measured to the nearest 0.1 cm with a fixed stadiometer. Whole body DEXA was performed to assess lean mass and inform energetic capacity calculations.

#### Patient-Reported Outcomes

Patient reported fatigue was measured using the Basic Fatigue Inventory (BFI). Quality of life, physical functioning, and breast cancer related symptoms were measured with the Functional Assessment of Cancer Therapy-Breast (FACT-B).

### Statistical Analyses

This is a single arm, longitudinal cohort pilot study to evaluate the feasibility. Feasibility was determined using all enrolled participants (n = 66). Sample size was driven by precision of estimation around secondary efficacy endpoints based on pilot data for peak power per kilogram lean mass generated in this population (17). Secondary efficacy endpoints were determined in the per protocol population, defined as those who completed all pre- and post-intervention assessments (n = 57). Categorical values were reported as frequencies and percentages in the evaluable population and continuous values were reported as means with standard deviation. The effect of the intervention on change in baseline power, steps per day, and patient-reported fatigue and quality of life were assessed using descriptive statistics. Paired t-tests were used to determine changes in effect estimates pre- and post-intervention in the secondary endpoints. In addition, patients were divided into tertiles of baseline energetic capacity by the Power Protocol-B, and differences in changes in endpoints between tertiles were determined using ANCOVA models, using Boneferonni to adjust for multiple comparisons. Pearson’s correlation was used to evaluate the relationship between changes in steps/day and changes in peak power per kg lean mass. SAS version 9.4 (SAS Institute, Cary, NC, USA) was used for analyses.

## Results

### Enrollment and Feasibility

**Figure 2:**
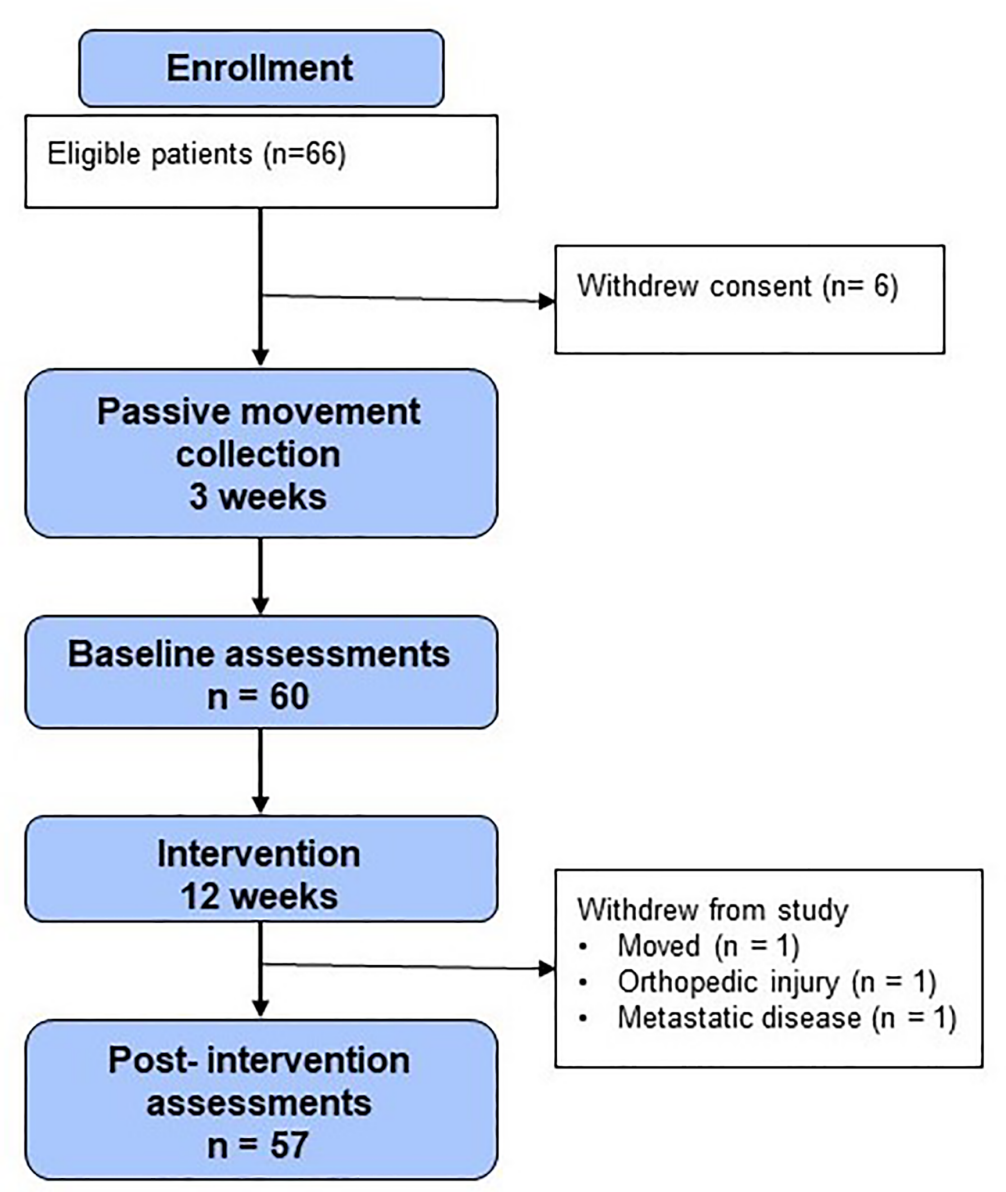
CONSORT diagram.

Sixty-six patients were enrolled in the study (**Figure 2**). Six patients withdrew consent prior to baseline procedures. Three patients who began the intervention did not complete the post-intervention procedures. Thus, 86% (n = 57) of participants in the enrolled population completed all study procedures pre- and post-intervention and were evaluable for the study endpoints. All of these patients (100%, n = 57) wore the GPS/accelerometer monitor for at least 10 waking hours per day, 4 days per week.

In addition to high levels of participation, the majority of feedback received from patients was positive. The majority of patients voiced comments such as, “kept me motivated”, and “enjoyed the study, made me more aware of exercise”. Many patients wished for this to be a standardized program, saying “If I could do it [the study] all over again, I would” and “good program every hospital should have”. All negative comments were surrounding the functionality of the watch: “I dislike the beep function”, “watch was too big and bulky”, and the limited functions available for activity: “would like to add more activities”, and “I want an additional nutritional component”.

Notably, there were no privacy concerns regarding GPS monitoring voiced by any approached or enrolled participant.

The 57 participants who completed both the baseline and post-intervention assessments are included in the analysis of the outcome measures. Baseline characteristics of these participants are shown in **Table 1**. Patients were an average of 59.2 ± 9.4 years old and 63.7 ± 58.6 months from initial breast cancer diagnosis. The majority had a history of stage I disease (53%) and had received chemotherapy (58%) and/or endocrine therapy (65%). The majority of patients were obese (53%) and sedentary, averaging 6,642 ± 2,817 steps/day during the baseline accelerometer data collection.

**Table 1:** Baseline characteristics of the evaluable population.

Characteristic	N = 57
**Age, mean (SD)**	59.2 (9.4)
**Body fat %, mean (SD)**	43.4 (8.0)
**BMI (kg/m^2^), mean (SD)**	32.6 (13.2)
**BMI category**	
** Normal**	8 (14)
** Overweight**	19 (33)
** Obese**	30 (53)
**Steps per day, mean (SD)**	6642 (2817)
**Steps per day category**	
** <5,000**	16 (28)
** 5,000–9,999**	35 (61)
** ≥10,000**	6 (11)
**Race**	
** Non-Hispanic white**	33 (58)
** Black**	16 (28)
** Hispanic white**	1 (2)
** Asian**	0 (0)
** Other**	7 (12)
**Education**	
** High school**	2 (4)
** Some college**	16 (28)
** College degree**	26 (46)
** Graduate degree**	13 (23)
**Income**	
** Not enough to be comfortable**	5 (9)
** Comfortable**	35 (61)
** More than comfortable**	16 (28)
** No answer**	1 (2)
**Employment status**	
** Part-time**	8 (14)
** Full-time**	27 (47)
** Retired**	15 (26)
** Disabled**	1 (2)
** Other**	6 (11)
**Time since diagnosis, months**	
**Mean (SD)**	63.7 (58.6)
**Stage**	
** *In-situ***	2 (4)
** I**	30 (53)
** II**	18 (32)
** III**	7 (12)
**Breast cancer subtype**	
** ER+/HER2−**	32 (56)
** ER+/HER2+**	6 (11)
** ER−/HER2+**	2 (4)
** ER−/HER2−**	17 (30)
**Treatment**	
** Surgery**	57 (100)
** Radiation**	38 (67)
** Chemotherapy**	33 (58)
** HER2 targeted therapy**	8 (14)
** Anti-estrogen therapy**	37 (65)

All data presented as No. (%) unless otherwise indicated.

BMI, body mass index; SD, standard deviation.

### Change in Steps Per Day and Energetic Capacity

As shown in **Table 2**, in the total population, average daily steps increased from 6,642 during the pre-intervention period, to 7,200 during the intervention (p = 0.01). Following the intervention, the total population improved energetic capacity significantly, with peak power normalized to lean body mass (W/kg) increasing the mean by 13.1% (p = 0.01). There was a moderate positive correlation between increase in steps/day during the intervention and increase in power generation capability on the stationary bicycle (r = 0.3, p = 0.03).

**Table 2:** Comparison of energy expenditure and energetic capacity at baseline and post-intervention.

Group*****	Energy expenditure (average steps/day)****			****Energetic capacity (peak power W/kg lean body mass)		
	Baseline	Post-intervention	p-value	Baseline	Post-intervention	p-value
**Total population**	6,642 (2,817)	7,200 (2,744)	**0.01**	1.76 (0.87)	1.99 (1.01)	**0.01**
**Low**	4,904 (2,722)	5,101 (2,595)	0.48	0.76 (0.28)	1.12 (0.43)	**<0.01**
**Moderate**	6,675 (1,831)	7,236 (1,863)	0.14	1.75 (0.21)	1.88 (0.48)	0.20
**High**	8,346 (2,771)	9,022 (2,254)	0.05	2.75 (0.40)	2.96 (0.98)	0.29

*Low, moderate, and high refer to tertile of baseline energetic capacity.

All values described as mean (standard deviation).

p < 0.05 is statistically significant, indicated by bold face type.

The mean baseline functional capacity measured in power/kg lean mass in the total evaluable population was 1.76 W/kg (SD 0.87), increasing to 1.99 W/kg (SD 1.01) post-intervention (p = 0.01). This is numerically higher than what was seen in our prior observational study of patients newly diagnosed with early stage breast cancer (1.55 W/kg, SD 0.88) (17), and lower than predicted W/kg of women regularly participating in endurance exercise (3.4 W/kg in 25 year old, 2.4 W/kg in 65 year olds) (23). Given the large variability in the baseline energetic capacity of the total population, additional exploratory analysis was done separating participants into tertiles based upon their baseline function (n = 19 in each group). Data for the total population and each tertile is displayed in **Table 2**. The lowest performing tertile at baseline had the largest and most significant improvement following the intervention, increasing the mean energetic capacity by 47% (p < 0.01); however, there was not a significant difference when comparing between group differences in each tertile (p = 0.65). All tertiles increased energy expenditure during the intervention, with a significant difference when comparing tertiles (p = 0.03). The low *versus* high tertiles were significantly different while adjusting for multiple comparisons (p = 0.02).

During the exploratory post-intervention period when subjects were no longer involved in the study intervention, adherence with wearing the activity monitor (defined as an average of 10 h per day, at least 4 days per week) fell to 78.9% (n = 45) of participants. Of those who continued to wear the monitor post-intervention, only 33% (n = 15) maintained energy expenditure in steps taken/day that were higher than baseline levels.

### Change in Patient-Reported Fatigue and Quality of Life

Patient-reported outcomes in the total evaluable population are displayed in **Table 3**. Following the intervention, quality of life by the FACT-B trial outcome index (TOI), which incorporates physical and functional well-being and breast cancer specific symptoms, improved significantly (p = 0.02). While this represents a statistically significant change, it is questionable whether this is a clinically important difference. Prior work suggests that even small improvements in FACT outcomes can be meaningful (24); however, a combined and anchor-based approach to determine the minimally important difference for the FACT-B TOI suggests it is 5 points, greater than what was seen in this study (25). Global fatigue scores by BFI did not change significantly (p = 0.16). Analysis by tertile of baseline function revealed the largest improvement in quality of life occurred in the highest functioning tertile, who also started with higher quality of life scores (73.7 ± 9.1 baseline *versus* 76.3 ± 9.3 post, p = 0.02). However, differences in changes between tertiles were not statistically significant (p = 0.70).

**Table 3:** Comparison of patient reported outcomes at baseline and post-intervention.

Element	Baseline	Post-intervention	P value
**FACT-B**			
Trial Outcome Index (total)	72.8 (10.1)	74.8 (10.4)	0.02
Physical well being	24.2 (3.3)	24.8 (3.3)	0.02
Emotional well being	20.4 (3.3)	20.8 (3.2)	0.15
Functional well being	23.3 (3.9)	23.7 (3.5)	0.30
Breast cancer symptom score	25.3 (5.2)	26.4 (5.5)	0.03
**Basic Fatigue Inventory**			
Global Fatigue Score	2.2 (1.9)	1.9 (1.9)	0.16

## Discussion

Physical function and physical activity are prognostic of increased mortality after breast cancer (4, 26). Increased risk of cardiovascular disease, metabolic syndrome, and recurrent breast cancer are compounded by the high rates of physical inactivity and deconditioning reported in this population (27–30), including the participants enrolled in this study. Here, we demonstrate feasibility of a 12-week intervention of personalized, wearable technology-based, dynamic physical activity prescriptions, derived from individual environment, movement patterns, and baseline functional capacity. Participants were highly adherent, with 95% (n = 57/60) of participants who began the intervention completing all assessments and wearing the GPS/accelerometer monitor for at least 10 waking hours/day, 4 days/week during the intervention. This approach was impactful, significantly improving both exercise capacity and daily movement in steps per day in breast cancer survivors.

In exploratory analyses, the most significant improvement in functional energetic capacity following the activity intervention occurred in the participants with the lowest capacity at baseline. This is the population least likely to have either the ability or motivation to enter or be successful in formalized exercise programs, and the most likely to benefit from gradual, personalized approaches. There remains a need for interventions that are approachable and deliverable to deconditioned patients who may lack the self-efficacy, ability, or comfort required for uptake of supervised, more intensive exercise programs. The gradual, personalized movement intervention described here may be a more accessible means to improve function and activity levels in these patients; additionally, this approach may serve as “prehabilitation” to improve function prior to a supervised or more intensive exercise intervention, a strategy we will evaluate in future work.

Our results align with multiple other physical activity and exercise interventions of variable design demonstrating feasibility and improved physical functioning and quality of life in this population. Many of these interventions involve supervised, moderate to high intensity aerobic and/or resistance exercise. While more intensive interventions likely achieve greater effect size in clinically relevant endpoints, these are difficult to generalize as a significant portion of the cancer survivor population cannot or will not participate. As multiple prior studies have shown, cost and access are barriers to participation in supervised interventions, and the majority of cancer survivors prefer home-based exercise (11). Unsupervised, home-based interventions may compromise the adherence seen in supervised programs; however, the use of digital technology to remotely monitor participation, provide real-time feedback, and engage patients may diminish this limitation (31). Furthermore, GPS/accelerometer delivered interventions such as used here translate objective data into motivation and goals for participants, promoting patient engagement without direct supervision. This may enhance self-efficacy, a known predictor of exercise adherence in cancer survivors (32, 33).

A novel strength of this study is the collection of detailed data from both accelerometer and GPS to provide granular movement information that is comprehensive and objective. To date, much research on physical activity behaviors or interventions in cancer survivors has been characterized by patient self-report, with variable reliability. Such approaches may not provide reliable, quantifiable metrics in deconditioned populations, and do not capture light intensity or “daily life” movements, as these are not typically thought of or recalled as physical activity. However, this general activity reduces sedentary time and impacts health and cancer specific outcomes (34–36). Several studies indicate that time spent in activity, rather than activity intensity, drives mortality benefit (37). Importantly, sedentary time and light physical activity do not appear to impact breast cancer incidence (38, 39); therefore, it is likely imperative to work toward moderate and vigorous activity as a long-term goal to impact recurrence in breast cancer survivors. While many studies have used accelerometers for physical activity data collection, few have used this in combination with GPS data in order to inform a dynamic intervention (40). The movement data collected during the intervention period was used not only as an efficacy endpoint, but to actually inform the intervention and personalize activity recommendations. This data was used to create activity prescriptions at the individual, rather than cohort, level. Using GPS and geographical information systems, activity was prescribed within the context of the patient’s habituated environment. It is hypothesized that this approach proved effective by removing traditional time and access barriers to activity, and may be more sustainable by teaching patients how to be more active within their normal environment. Only three patients out of 66 eligible felt the study intervention was too time intensive for participation. When monitoring movement data after the intervention, we observed a large portion of participants no longer tracking their behaviors in the absence of active intervention. Furthermore, of those that did continue to wear the activity monitor, behavior change was not sustained in the majority of participants. While this is observational, it is hypothesized that strategies utilizing continuous intervention will result in the most sustained activity changes in this population.

Several limitations of this study will further inform future directions. The study is non-randomized; it is possible that improvements seen are simply a result of healthcare team attention, rather than the activity intervention. Furthermore, improvements in power generation may be simply a result of familiarity with the study procedures. However, our prior work evaluating change in power generation after primary breast cancer therapy in the absence of intervention demonstrated a significant decline in power over 6 months (17). The small sample size and single arm design does not allow for investigation of demographic or clinical characteristics that may predict greater benefit to this approach. In addition, the intervention required personnel expertise and was not fully automated. We are currently utilizing a machine learning approach to successfully automate all intervention procedures to improve scalability. A future, randomized trial will evaluate an automated version of this intervention compared to a usual care control arm with activity monitors alone. This will be enriched for the lowest functioning patients not currently participating in regular physical activity, and will focus on phenotypic moderators of this approach. Based on patient feedback, future iterations of this intervention will also include more activities than walking/running, and will allow use of other wearable devices.

This study designed to encourage breast cancer survivors to increase physical activity within their typical environment was feasible with high patient engagement, successfully increasing energy expenditure and, in turn, functional capacity. High variability in both baseline activity and function with more significant improvements in the most deconditioned patients supports the need for work focused on precision approaches to physical activity interventions. Future work should utilize accelerometer assessed physical activity and sedentary time, varying methods, dose, and delivery based on individual environment, lifestyle, and exercise capacity.

## Data Availability Statement

The raw data supporting the conclusions of this article will be made available by the authors, without undue reservation.

## Ethics Statement

The studies involving human participants were reviewed and approved by Indiana University Institutional Review Board. The patients/participants provided their written informed consent to participate in this study.

## Author Contributions

TB: conceptualization, methodology, investigation, formal analysis, and writing. SA: formal analysis and writing. TO: methodology, investigation, and data analysis. KM: funding acquisition, conceptualization, investigation, supervision, and writing (review and editing). JS: conceptualization, methodology, investigation, formal analysis, and writing. All authors contributed to the article and approved the submitted version.

## Funding

This research was supported by funding from Susan G. Komen (SAC150007, PI: KM).

## Conflict of Interest

TO reports non-financial support from iMETx Inc during the conduct of the study and from iMETx Inc outside the submitted work.

The remaining authors declare that the research was conducted in the absence of any commercial or financial relationships that could be construed as a potential conflict of interest.

## References

[1] Lahart IM Metsios GS Nevill AM Carmichael AR. Physical activity, risk of death and recurrence in breast cancer survivors: A systematic review and meta-analysis of epidemiological studies. Acta Oncol (2015) 54(5):635–54. 10.3109/0284186X.2014.998275 25752971

[2] Buffart LM Kalter J Sweegers MG Courneya KS Newton RU Aaronson NK. Effects and moderators of exercise on quality of life and physical function in patients with cancer: An individual patient data meta-analysis of 34 RCTs. Cancer Treat Rev (2017) 52:91–104. 10.1016/j.ctrv.2016.11.010 28006694

[3] Jones LW Courneya KS Mackey JR Muss HB Pituskin EN Scott JM. Cardiopulmonary function and age-related decline across the breast cancer survivorship continuum. J Clin Oncol (2012) 30(20):2530–7. 10.1200/JCO.2011.39 PMC3397786 22614980

[4] Braithwaite D Satariano WA Sternfeld B Hiatt RA Ganz PA Kerlikowske K. Long-term prognostic role of functional limitations among women with breast cancer. J Natl Cancer Inst (2010) 102(19):1468–77. 10.1093/jnci/djq344 PMC2950169 20861456

[5] Lucas AR Levine BJ Avis NE. Posttreatment trajectories of physical activity in breast cancer survivors. Cancer (2017) 123(14):2773–80. 10.1002/cncr.30641 PMC5498227 28272835

[6] Irwin ML Crumley D McTiernan A Bernstein L Baumgartner R Gilliland FD. Physical activity levels before and after a diagnosis of breast carcinoma: the Health, Eating, Activity, and Lifestyle (HEAL) study. Cancer (2003) 97(7):1746–57. 10.1002/cncr.11227 PMC3034406 12655532

[7] Dieli-Conwright CM Courneya KS Demark-Wahnefried W Sami N Lee K Sweeney FC. Aerobic and resistance exercise improves physical fitness, bone health, and quality of life in overweight and obese breast cancer survivors: a randomized controlled trial. Breast Cancer Res (2018) 20(1):124. 10.1186/s13058-018-1051-6 30340503 PMC6194749

[8] Dieli-Conwright CM Courneya KS Demark-Wahnefried W Sami N Lee K Buchanan TA. Effects of Aerobic and Resistance Exercise on Metabolic Syndrome, Sarcopenic Obesity, and Circulating Biomarkers in Overweight or Obese Survivors of Breast Cancer: A Randomized Controlled Trial. J Clin Oncol (2018) 36(9):875–83. 10.1200/JCO.2017.75.7526 PMC5858524 29356607

[9] van Waart H van Harten WH Buffart LM Sonke GS Stuiver MM Aaronson NK. Why do patients choose (not) to participate in an exercise trial during adjuvant chemotherapy for breast cancer? Psychooncology (2016) 25(8):964–70. 10.1002/pon.3936 26282696

[10] Kampshoff CS Jansen F van Mechelen W May AM Brug J Chinapaw MJ. Determinants of exercise adherence and maintenance among cancer survivors: a systematic review. Int J Behav Nutr Phys Act (2014) 11:80. 10.1186/1479-5868-11-80 24989069 PMC4096543

[11] Hardcastle SJ Maxwell-Smith C Kamarova S Lamb S Millar L Cohen PA. Factors influencing non-participation in an exercise program and attitudes towards physical activity amongst cancer survivors. Support Care Cancer (2018) 26(4):1289–95. 10.1007/s00520-017-3952-9 29090387

[12] Peters D Calvo RA Ryan RM. Designing for Motivation, Engagement and Wellbeing in Digital Experience. Front Psychol (2018) 9:797. 10.3389/fpsyg.2018.00797 29892246 PMC5985470

[13] Sullivan AN Lachman ME. Behavior Change with Fitness Technology in Sedentary Adults: A Review of the Evidence for Increasing Physical Activity. Front Public Health (2016) 4:289. 10.3389/fpubh.2016.00289 28123997 PMC5225122

[14] Tcymbal A Demetriou Y Kelso A Wolbring L Wunsch K Wasche H. Effects of the built environment on physical activity: a systematic review of longitudinal studies taking sex/gender into account. Environ Health Prev Med (2020) 25(1):75. 10.1186/s12199-020-00915-z 33246405 PMC7697377

[15] Beyer KMM Szabo A Hoormann K Stolley M. Time spent outdoors, activity levels, and chronic disease among American adults. J Behav Med (2018) 41(4):494–503. 10.1007/s10865-018-9911-1 29383535 PMC6031452

[16] Piercy KL Troiano RP Ballard RM Carlson SA Fulton JE Galuska DA. The Physical Activity Guidelines for Americans. JAMA (2018) 320(19):2020–8. 10.1001/jama.2018.14854 PMC9582631 30418471

[17] Ballinger TJ Reddy A Althouse SK Nelson EM Miller KD Sledge JS. Impact of primary breast cancer therapy on energetic capacity and body composition. Breast Cancer Res Treat (2018). 10.1007/s10549-018-4924-6 PMC6208924 30136009

[18] Charmaz K. Constructing grounded theory. London: Sage (2014).

[19] Lave J. Situated learning: legitimate peripheral participation. New York, NY: Cambridge University Press (1991).

[20] Fuller D Colwell E Low J Orychock K Tobin MA Simango B. Reliability and Validity of Commercially Available Wearable Devices for Measuring Steps, Energy Expenditure, and Heart Rate: Systematic Review. JMIR Mhealth Uhealth (2020) 8(9):e18694. 10.2196/18694 32897239 PMC7509623

[21] Troiano RP Berrigan D Dodd KW Masse LC Tilert T McDowell M. Physical activity in the United States measured by accelerometer. Med Sci Sports Exerc (2008) 40(1):181–8. 10.1249/mss.0b013e31815a51b3 18091006

[22] Carrel AL Sledge JS Ventura SJ Clark RR Peterson SE Eickhoff JC. Measuring aerobic cycling power as an assessment of childhood fitness. J Strength Cond Res (2008) 22(1):192–5. 10.1519/JSC.0b013e31815f9ca7 18296974

[23] Ballinger TJ Reddy A Althouse SK Nelson EM Miller KD Sledge JS. Impact of primary breast cancer therapy on energetic capacity and body composition. Breast Cancer Res Treat (2018) 172(2):445–52. 10.1007/s10549-018-4924-6 PMC6208924 30136009

[24] Brown SJ Ryan HJ Brown JA. Age-Associated Changes In VO2 and Power Output - A Cross-Sectional Study of Endurance Trained New Zealand Cyclists. J Sports Sci Med (2007) 6(4):477–83. PMC3794488 24149481

[25] Cella D Hahn EA Dineen K. Meaningful change in cancer-specific quality of life scores: differences between improvement and worsening. Qual Life Res (2002) 11(3):207–21. 10.1023/a:1015276414526 12074259

[26] Eton DT Cella D Yost KJ Yount SE Peterman AH Neuberg DS. A combination of distribution- and anchor-based approaches determined minimally important differences (MIDs) for four endpoints in a breast cancer scale. J Clin Epidemiol (2004) 57(9):898–910. 10.1016/j.jclinepi.2004.01.012 15504633

[27] Sehl M Lu X Silliman R Ganz PA. Decline in physical functioning in first 2 years after breast cancer diagnosis predicts 10-year survival in older women. J Cancer Surviv (2013) 7(1):20–31. 10.1007/s11764-012-0239-5 23232922 PMC3568656

[28] Gernaat SAM Boer JMA van den Bongard DHJ Maas A van der Pol CC Bijlsma RM. The risk of cardiovascular disease following breast cancer by Framingham risk score. Breast Cancer Res Treat (2018) 170(1):119–27. 10.1007/s10549-018-4723-0 PMC5993849 29492735

[29] Koene RJ Prizment AE Blaes A Konety SH. Shared Risk Factors in Cardiovascular Disease and Cancer. Circulation (2016) 133(11):1104–14. 10.1161/CIRCULATIONAHA.115.020406 PMC4800750 26976915

[30] Uzer G Thompson WR Sen B Xie Z Yen SS Miller S. Cell Mechanosensitivity to Extremely Low-Magnitude Signals Is Enabled by a LINCed Nucleus. Stem Cells (2015) 33(6):2063–76. 10.1002/stem.2004 PMC4458857 25787126

[31] Mason C Alfano CM Smith AW Wang CY Neuhouser ML Duggan C. Long-term physical activity trends in breast cancer survivors. Cancer Epidemiol Biomarkers Prev (2013) 22(6):1153–61. 10.1158/1055-9965.EPI-13-0141 PMC3688258 23576689

[32] Lyons EJ Lewis ZH Mayrsohn BG Rowland JL. Behavior change techniques implemented in electronic lifestyle activity monitors: a systematic content analysis. J Med Internet Res (2014) 16(8):e192. 10.2196/jmir.3469 25131661 PMC4147713

[33] Kampshoff CS van Mechelen W Schep G Nijziel MR Witlox L Bosman L. Participation in and adherence to physical exercise after completion of primary cancer treatment. Int J Behav Nutr Phys Act (2016) 13(1):100. 10.1186/s12966-016-0425-3 27612561 PMC5016937

[34] McAuley E Blissmer B. Self-efficacy determinants and consequences of physical activity. Exerc Sport Sci Rev (2000) 28(2):85–8. 10902091

[35] LaMonte MJ Lewis CE Buchner DM Evenson KR Rillamas-Sun E Di C. Both Light Intensity and Moderate-to-Vigorous Physical Activity Measured by Accelerometry Are Favorably Associated With Cardiometabolic Risk Factors in Older Women: The Objective Physical Activity and Cardiovascular Health (OPACH) Study. J Am Heart Assoc (2017) 6(10). 10.1161/JAHA.117.007064 PMC5721888 29042429

[36] Ehlers DK Fanning J Salerno EA Aguinaga S Cosman J Severson J. Replacing sedentary time with physical activity or sleep: effects on cancer-related cognitive impairment in breast cancer survivors. BMC Cancer (2018) 18(1):685. 10.1186/s12885-018-4603-3 29940894 PMC6019533

[37] Phillips SM Awick EA Conroy DE Pellegrini CA Mailey EL McAuley E. Objectively measured physical activity and sedentary behavior and quality of life indicators in survivors of breast cancer. Cancer (2015) 121(22):4044–52. 10.1002/cncr.29620 PMC4635035 26308157

[38] Matthews CE Keadle SK Troiano RP Kahle L Koster A Brychta R. Accelerometer-measured dose-response for physical activity, sedentary time, and mortality in US adults. Am J Clin Nutr (2016) 104(5):1424–32. 10.3945/ajcn.116.135129 PMC5081718 27707702

[39] Kobayashi LC Janssen I Richardson H Lai AS Spinelli JJ Aronson KJ. A case-control study of lifetime light intensity physical activity and breast cancer risk. Cancer Causes Control (2014) 25(1):133–40. 10.1007/s10552-013-0312-z 24158779

[40] Nomura SJO Dash C Sheppard VB Bowen D Allison M Barrington W. Sedentary time and postmenopausal breast cancer incidence. Cancer Causes Control (2017) 28(12):1405–16. 10.1007/s10552-017-0968-x PMC5687985 28975422

[41] Schaffer K Panneerselvam N Loh KP Herrmann R Kleckner IR Dunne RF. Systematic Review of Randomized Controlled Trials of Exercise Interventions Using Digital Activity Trackers in Patients With Cancer. J Natl Compr Canc Netw (2019) 17(1):57–63. 10.6004/jnccn.2018.7082 30659130 PMC6519727

